# A Time-Course Comparison of Skeletal Muscle Metabolomic Alterations in Walker-256 Tumour-Bearing Rats at Different Stages of Life

**DOI:** 10.3390/metabo11060404

**Published:** 2021-06-20

**Authors:** Gabriela de Matuoka e Chiocchetti, Leisa Lopes-Aguiar, Natália Angelo da Silva Miyaguti, Lais Rosa Viana, Carla de Moraes Salgado, Ophelie Ocean Orvoën, Derly Florindo, Rogério Williams dos Santos, Maria Cristina Cintra Gomes-Marcondes

**Affiliations:** 1 Laboratory of Nutrition and Cancer, Department of Structural and Functional Biology, Biology Institute, University of Campinas (UNICAMP), Rua Monteiro Lobato, 255, Campinas 13083862, SP, Brazil; leisaaguiar@yahoo.com.br (L.L.-A.); namiyaguti@gmail.com (N.A.d.S.M.); lala.viana311088@gmail.com (L.R.V.); carlamsalgado@gmail.com (C.d.M.S.); ophelie.orv@gmail.com (O.O.O.); dede.flor2011@gmail.com (D.F.); rowisa@unicamp.br (R.W.d.S.); 2 Biology Department, University of Angers, 49000 Anger, France

**Keywords:** cachexia, metabolomics, skeletal muscle, time-course, Walker-256 tumour

## Abstract

Cancer cachexia is a severe wasting condition that needs further study to find ways to minimise the effects of damage and poor prognosis. Skeletal muscle is the most impacted tissue in cancer cachexia; thus, elucidation of its metabolic alterations could provide a direct clue for biomarker research and be applied to detect this syndrome earlier. In addition, concerning the significant changes in the host metabolism across life, this study aimed to compare the metabolic muscle changes in cachectic tumour-bearing hosts at different ages. We performed ^1^H-NMR metabolomics in the gastrocnemius muscle in weanling and young adult Walker-256 tumour-bearing rats at different stages of tumour evolution (initial, intermediate, and advanced). Among the 49 metabolites identified, 24 were significantly affected throughout tumour evolution and 21 were significantly affected regarding animal age. The altered metabolites were mainly related to increased amino acid levels and changed energetic metabolism in the skeletal muscle, suggesting an expressive catabolic process and diverted energy production, especially in advanced tumour stages in both groups. Moreover, these changes were more severe in weanling hosts throughout tumour evolution, suggesting the distinct impact of cancer cachexia regarding the host’s age, highlighting the need to adopting the right animal age when studying cancer cachexia.

## 1. Introduction

Cancer cachexia is a multi-factorial syndrome associated with skeletal muscle wasting with or without adipose tissue loss, chronic systemic inflammation, anorexia, poor prognosis, and reduced survival [1]. Depending on the type and stage of cancer, up to 80% of all patients are affected by this syndrome [2], which is responsible for approximately 30% of all cancer-related deaths [3].

Cachexia is also considered to be a multi-organ syndrome, since it leads to multiple organ metabolism changes such as in the heart, liver, brain, white and brown adipose tissue, and especially skeletal muscle tissue, the most affected one [4]. Fearon and colleagues have described that cachexia has three stages: precachexia, cachexia, and refractory cachexia [5]. The progression of this syndrome is related to multiple factors, including systemic inflammation, cancer stage, anorexia, and response to anti-cancer treatment. In the refractory stage, weight loss management is not possible due to the intensive catabolism related to the presence of cachectic factors and, so far, there is no effective treatment for refractory cachexia [5,6]. Thus, it is critically important to identify the onset of cachexia earlier and develop molecular interventions to reduce or delay the progression of this disease [7].

As cachexia progresses, proteolysis of myofibrillar proteins increases [8]. This causes the release of amino acids that can be used for energy production via the tricarboxylic acid (TCA) cycle in the muscle [9] and for hepatic synthesis of the acute-phase response proteins. Furthermore, some released amino acids, such as alanine, and other metabolites, such as glycerol from lipolysis and lactate from glycolysis, could be converted into glucose by liver gluconeogenesis, favouring tumour growth [10]. Additionally, during cachexia, decreased energy and growth signalling, as well as increased content of pro-inflammatory cytokines, such as interleukin-6 and tumour necrosis factor-alpha, downregulate the activity of the mechanistic target of rapamycin [11], which is one of the most important regulators of protein, nucleotide, and lipid metabolism. Better elucidation of all these metabolic alterations contributes to managing the agents and cause(s) of cachexia, allowing the development of possible treatments. Along these lines, omics approaches have become more popular for studying metabolic alterations during cachexia [12]. The impact of cancer cachexia is likely to be distinct at different ages, given the specific challenges of each development stage. To our knowledge, no study so far has compared the muscle metabolic changes in cachectic tumour-bearing hosts at different stages of life. Therefore, this study aimed to compare the muscle metabolomic alterations, in a time-course approach, between weanling and young adult Walker-256 tumour-bearing rats during the different stages of tumour evolution.

## 2. Results

### 2.1. Walker-256 Tumour Growth Induced Cachexia, Leading to Loss of Body Weight, Spoliation of the Gastrocnemius Muscle, and Nitrogen Imbalance at Both Ages

**Figure 1:**
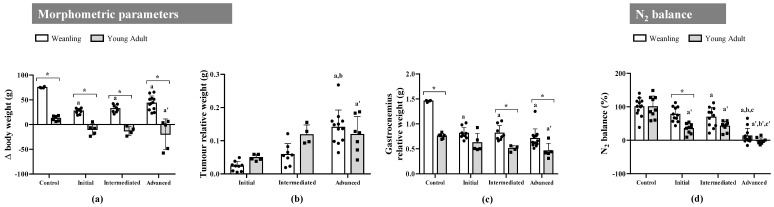
Morphometric parameters and N_2_ balance identified in weanling and young adult female Walker-256 tumour-bearing rats. (**a**) Δ body weight, (**b**) relative tumour weight, (**c**) relative gastrocnemius weight, and (**d**) N_2_ balance, distributed into control (WC and AC) and initial (WWi and AWi), intermediate (WWm and AWm), and advanced (WWa and AWa) Walker tumour-bearing animals. Legend: Δ body weight was calculated as [final body weight−initial body weight] for non-tumour-bearing groups and as [(final body weight−tumour weight)−initial body weight] for tumour-bearing groups. The relative values were calculated by dividing the tumour and gastrocnemius muscle weights by each animal’s respective initial body weight. N_2_ balance was calculated as [food intake N_2_−(urinary N_2_ + faecal N_2_)]. Data were expressed as means ± standard deviation and analysed by two-way ANOVA, followed by post-hoc Tukey’s honestly significant difference. *p*-value < 0.05 in comparison with the (a) WC, (b) WWi, and (c) WWm groups; with the (a’) AC, (b’) AWi, and (c’) AWm groups; and with the young adult (*) group.

Among the morphometric parameters (Supplementary Table S1), we observed that tumours negatively affected body weight gain in the weanling tumour-bearing groups (WW) when compared with the weanling non-tumour-bearing group (WC) (reduction around 50% at the endpoint). There was no statistical difference in WW body weight during tumour evolution. Within the young adult tumour-bearing groups (AW), only the advanced tumour-bearing animals (AWa) had a significantly different variation in body weight when compared with the young adult non-tumour-bearing group (AC). Weanling animals differed from young adults in the controls and at all tumour stages (Figure 1a).

In the young adult tumour-bearing animals, the AWa group had a significantly higher tumour relative weight when compared with initial tumour-bearing animals (AWi), and tumour weight was similar to that in intermediate tumour-bearing animals (AWm). In the weanling group, the relative tumour size was higher in the advanced tumour animals (WWa) than the initial tumour (WWi) and intermediate tumour (WWm) animals. However, there was no difference in the relative tumour size between WW and AW animals (Figure 1b).

Regarding the relative gastrocnemius muscle weight, all tumour-bearing animals in the weanling group presented a significant reduction when compared with WC animals. In the young adult groups, only AWa animals had a reduction in the gastrocnemius weight when compared with the AC group. Considering the body development in weanling animals, we observed a significantly higher relative gastrocnemius weight in the WC, WWm, and WWa groups compared with the respective young adult groups (Figure 1c).

Concerning the nitrogen balance, we observed a significant reduction in the intermediate and advanced tumour-bearing groups, independent of age, compared with non-tumour-bearing rats. The initial tumour stage was significantly different only in young adult groups compared with the AC group. Weanling animals differed from young adults in the initial tumour stage only (Figure 1d).

### 2.2. Cachexia Induces a Disorder in the Gastrocnemius Muscle’s Metabolomic Profile in Weanling and Young Adult Rats

**Figure 2:**
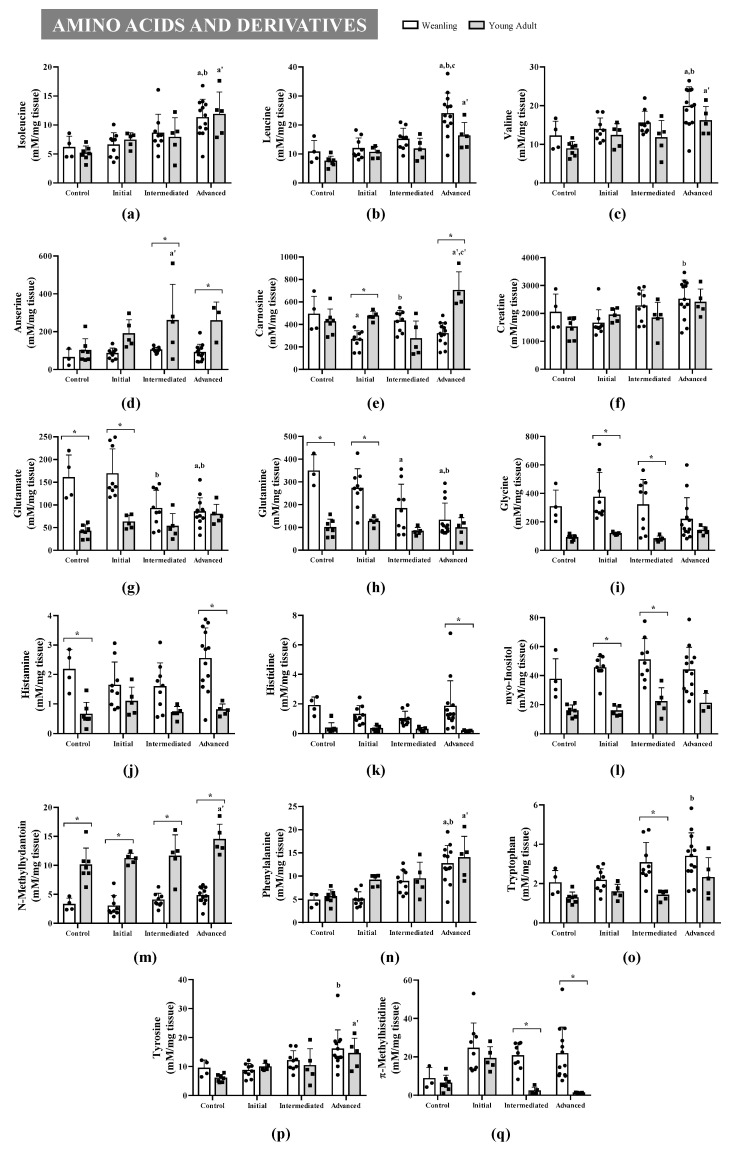
Gastrocnemius muscles’ metabolic profile of amino acids and derivatives identified in weanling and young adult female Walker-256 tumour-bearing rats. Concentration of BCAAs: (**a**) isoleucine, (**b**) leucine, (**c**) valine, (**d**) anserine, (**e**) carnosine, (**f**) creatine, (**g**) glutamate, (**h**) glutamine, (**i**) glycine, (**j**) histamine, (**k**) histidine, (**l**) myo-inositol, (**m**) N-methyl-hydantoin, (**n**) phenylalanine, (**o**) tryptophan, (**p**) tyrosine, and (**q**) π-methylhistidine metabolites distributed into weanling and young adult control (WC and AC) and initial (WWi and AWi), intermediate (WWm and AWm) and advanced (WWa and Awa) Walker tumour-bearing animals. Legend: Data are expressed as means ± standard deviation and analysed by two-way ANOVA, followed by post-hoc Tukey’s honestly significant difference. *p*-value < 0.05 in comparison with (a) WC, (b) WWi, and (c) WWm groups; with (a’) AC, (b’) AWi, and (c’) AWm groups; and with young adult (*) groups.

**Figure 3:**
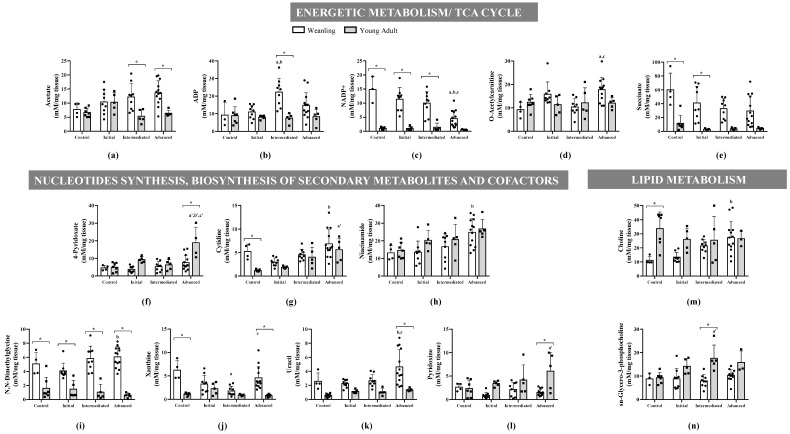
Gastrocnemius muscles’ metabolic profile of energetic metabolism (**a**–**e**), nucleotide synthesis and biosynthesis of secondary metabolites and cofactors (**f**–**l**), and lipid metabolism (**m**,**n**) identified in young and adult female Walker-256 tumour-bearing rats. Concentration of (**a**) acetate, (**b**) ADP, (**c**) NADP+, (**d**) o-acetylcarnitine, (**e**) succinate, (**f**) 4-pyridoxate, (**g**) cytidine, (**h**) niacinamide, (**i**) N,N-dimethylglycine, (**j**) xanthine, (**k**) uracil, (**l**) pyridoxine, (**m**) choline, (**n**) sn-glycero-3-phosphocholine metabolites distributed into weanling and young adult control (WC and AC) and initial (WWi and AWi), intermediate (WWm and AWm), and advanced (WWa and Awa) Walker tumour-bearing rats. Legend: Data are expressed as means ± standard deviation and analysed by two-way ANOVA, followed by post-hoc Tukey’s honestly significant difference. *p*-value < 0.05 in comparison with (a) WC, (b) WWi, and (c) WWm groups; with (a’) AC, (b’) AWi, and (c’) AWm groups; and with young adult (*) groups.

**Figure 4:**
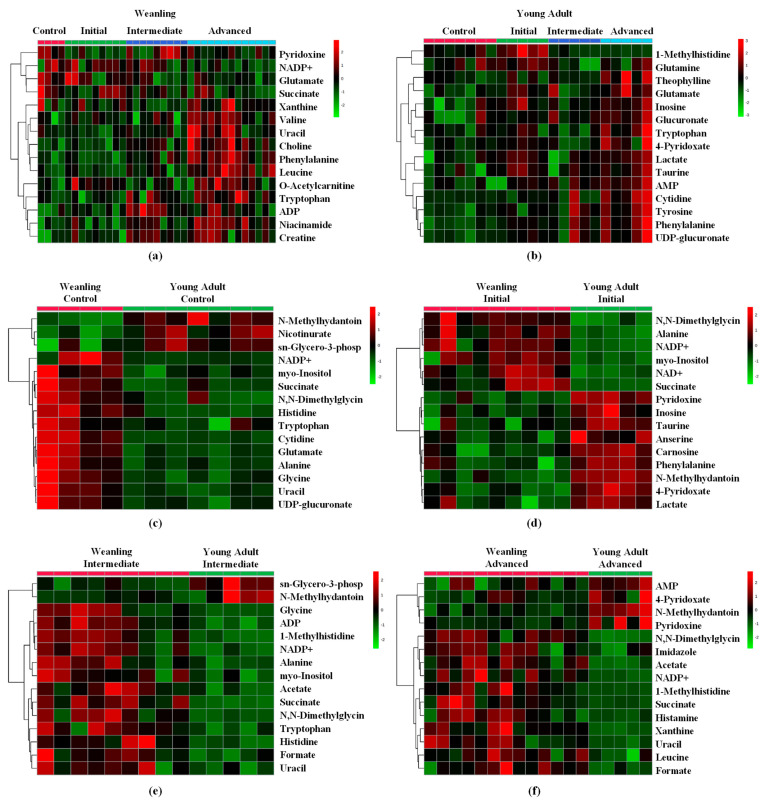
Heatmaps of gastrocnemius muscle identified in weanling and young adult female Walker-256 tumour-bearing rats. Concentrations of the 15 most important metabolites in (**a**) weanling and (**b**) young adult rats throughout tumour evolution (control—WC and AC; initial—WWi and AWi; intermediate—WWm and AWm; advanced—WWa and AWa) and regarding animal age (weanling and young adult) in (**c**) WC and AC, (**d**) WWi and AWi, (**e**) WWm and AWm, and (**f**) WWa and AWa Walker tumour-bearing rats. Legend: Data are expressed as abundance ratios (high: red and low: green) of the corresponding metabolites.

Among the 49 gastrocnemius muscle metabolites identified (Supplementary Figure S1 and Table S2), 24 were significantly affected throughout tumour evolution (initial, intermediate, and advanced) and 21 were significantly affected regarding animal age (weanling and young adult) (Figure 2 and Figure 3). The altered metabolites were grouped into four categories—amino acids and derivatives (Figure 2), energetic metabolism/TCA cycle, nucleotides synthesis, biosynthesis of secondary metabolites and cofactors, and lipid metabolism (Figure 3)—to guide the discussion. These alterations can also be observed in the heatmaps, where data show the difference in the metabolite concentration between the weanling and young adult groups (Figure 4).

#### 2.2.1. Metabolites Are Affected throughout Tumour Evolution in Weanling and Young Adult Tumour-Bearing Animals

In the amino acids and derivatives category, in the weanling groups, seven metabolites were significantly increased due to tumour effects (isoleucine, valine, and phenylalanine: WWa > WC and WWi; leucine: WWa > WC, WWi, and WWm; creatine, tryptophan, and tyrosine: WWa > WWi) and three metabolites were significantly decreased (carnosine: WWi < WC; glutamate: WWm < WWi and WWa < WC and WWi; glutamine: WWm < WC and WWa < WC and WWi). Meanwhile, in the young adult groups, only eight metabolites were significantly increased (isoleucine, leucine, valine, N-methyl-hydantoin, phenylalanine, and tyrosine: AWa > AC; anserine: AWm > AC; carnosine: AWa > AC and AWm) (Figure 2).

In the weanling group, regarding the energetic metabolism/TCA cycle, two metabolites significantly increased with tumour evolution (ADP: WWi > WC and/or WWi; and O-acetylcarnitine: WWa > WC and WWm) and two metabolites were significantly decreased (NADP+: WWa < WC, WWi, and WWm; xanthine: WWm < WC and WWa < WWm). In the nucleotide synthesis and biosynthesis of secondary metabolites and cofactors categories, four metabolites were significantly increased (uracil, cytidine, niacinamide, and N,N-dimethylglycine: WWa > WWi, and/or WWm; O-acetylcarnitine: WWa > WC and WWm) and in the lipid metabolism category, choline was increased (WWa > WWi and/or WWm). In young adult groups, four metabolites were significantly enhanced by the tumours’ effects (cytidine and pyridoxine: AWa > AC; sn-glycero-3-phosphocholine: AWm > AC; 4-pyridoxate: AWa > AC, AWi, and AWm) in these three categories (Figure 3).

Analysis of these changed metabolites showed that the most impacted pathways in WW groups affected by tumour evolution were aminoacyl-tRNA biosynthesis; valine, leucine, and isoleucine biosynthesis; phenylalanine, tyrosine, and tryptophan biosynthesis; nitrogen metabolism; and D-glutamine and D-glutamate metabolism. In the AW groups during tumour evolution, the most impacted pathways were aminoacyl-tRNA biosynthesis; valine, leucine, and isoleucine biosynthesis; phenylalanine, tyrosine, and tryptophan biosynthesis; and vitamin B6 metabolism (Table 1).

#### 2.2.2. Metabolites Affected Regarding the Age of the Tumour-Bearing Animals

In the amino acids and derivatives category, eight metabolites were significantly increased in weanlings and decreased in the young adult group (glutamate and glutamine: WC > AC and WWi > AWi; glycine and myo-inositol: WWi > AWi and WWm > AWm; histamine and histidine: WC > AC and/or WWa > AWa; tryptophan: WWm > AWm and π-methylhistidine: WWm > AWm and WWa > AWa), and three metabolites were significantly decreased in weanlings and increased in the young adult group (anserine: WWm < AWm and WWa < AWa; carnosine: WWi < AWi and WWa < AWa; N-methyl-hydantoin: < all comparisons) (Figure 2).

Regarding the energetic metabolism, five metabolites were significantly increased in weanlings and decreased in the young adult group (acetate: WWm > AWm and WWa > AWa; succinate: WC > AC and WWi > AWi; ADP (adenosine di-phosphate) WWm > AWm; NADP+ (nicotinamide adenine dinucleotide phosphate): WC > AC and WWi > AWi and WWm > AWm; and xanthine: WC > AC and WWa > AWa). In the nucleotide synthesis and biosynthesis of secondary metabolites and cofactors category, two metabolites were significantly increased in weanlings and decreased in the young adult group (uracil: WWa > AWa; N, N-dimethylglycine: > all comparisons) and only three metabolites were significantly decreased in weanlings and increased in the young adult group (sn-glycero-3-phosphocholine: WWm < AWm; pyridoxine and 4-pyridoxate: WWa < AWa) (Figure 3).

Analysing these altered metabolites, we observed that the pathways most impacted by the tumour effects in the WW group vs. the AW group were aminoacyl-tRNA biosynthesis, nitrogen metabolism, D-glutamine and D-glutamate metabolism, histidine metabolism, β-alanine metabolism, and glyoxylate and dicarboxylate metabolism (Table 1).

## 3. Discussion

In cancer cachexia, the mechanisms related to this severe wasting condition are still not fully understood [13,14]. Several metabolomic studies into cancer cachexia were based on metabolomic analysis of the serum, giving a global view of the metabolic alterations induced by this syndrome in the host [15,16,17,18,19]. Despite their importance, they did not provide detailed data on muscle wasting itself. In this context, analysis of the skeletal muscle metabolites could provide a direct clue for biomarker research and also be applied for early detection of this syndrome [20]. Most of the preclinical cancer studies evaluated the metabolic muscle changes in cachectic tumour-bearing rats aged 8–12 weeks, representing an adult host [21,22,23]. The definition of “adult” in this context is likely to be related to the sexual maturity of the rodent, which is not a sufficient basis for considering the total development of the animal. Significant differences exist in disease-relevant systems between young and aged animals, and these differences may affect the outcome of studies investigating basic disease biology [24,25]. Moreover, the impact of cancer cachexia is likely to be distinct regarding age, given the challenges of each specific development stage [26]. To our knowledge, no study so far has compared the metabolic muscle changes at different ages in cachectic tumour-bearing hosts, showing these metabolic differences concerning the stage of life. Thus, this study performed a time-course comparison of metabolomic alterations in the gastrocnemius muscle in weanling and young adult hosts, and showed that 27 metabolites were significantly affected throughout tumour evolution.

Intense body weight loss is expected during cancer cachexia, as verified in the AW animals in this study, and similar to others’ results for this same tumour model [27,28,29]. The tumour also affected the body growth and development in WW animals, but to a lesser extent, probably because this group was still in the growing phase. Accordingly, the tumour growth accounted for the reduction in gastrocnemius mass in both the WW and AW groups, which showed intense spoliation in all tumour stages in WW rats but only in advanced tumours in the AW animals. Muscle homeostasis is maintained by a balance between the synthesis and degradation of muscle protein [30,31], and this homeostasis can be disrupted by cytokines and pro-cachectic mediators released by the tumour, its metastases, activated immune cells, and other tissues/organs, causing muscle wasting in the host [4,32]. Since skeletal muscles are a source of amino acids, this spoliation could be corroborated by a decreased nitrogen balance [33]. In accordance with this, our results showed a progressive nitrogen imbalance that accompanied tumour growth in both WW and AW animals. However, here, in the present work, we could not establish whether the decreased nitrogen balance associated with cachexia was from altered rates of synthesis and/or degradation in muscle tissue [34].

These alterations were accompanied by metabolic changes in the amino acids and derivates category in gastrocnemius tissue of both the WW and AW groups. Seven (the branched chain amino acids (BCAAs) isoleucine, leucine, and valine, and creatine, phenylalanine tryptophan, and tyrosine) and eight (BCAAs, N-methyl-hydantoin, phenylalanine, tyrosine, anserine, and carnosine) metabolite levels were augmented in WW and AW animals, respectively, where some of these alterations followed other studies [35,36]. Tseng and collaborators found an increase in free amino acids in cachectic gastrocnemius muscle, including BCAAs, and they concluded that this could indicate increased muscle protein breakdown [35]. Cui and collaborators found an increase in the metabolites glutamate and arginine, and in the BCAAs leucine and isoleucine in cachectic gastrocnemius tissue and concluded that some of these metabolites—especially the BCAAs—might either act as precursors to promote protein synthesis or be metabolised to replenish the TCA intermediates [36]. The BCAAs are central in the maintenance of lean body mass and regulation of skeletal muscle protein metabolism, since myofibrillar proteins are composed of ≈ 18% BCAAs [37]. Thus, the increase in BCAAs found in the gastrocnemius muscle of WW and AW animals could be associated with muscle protein breakdown.

Still regarding the amino acid category, we also found augmented phenylalanine in the WWa and AWa groups and an increase in tryptophan concentration only in the WWa group, likely indicating intense spoliation in these animals. Similarly, Lautaoja and collaborators [38] found phenylalanine and tryptophan to be augmented in the gastrocnemius of C26 tumour-bearing mice. Moreover, the authors revealed that the increased content of free phenylalanine was strongly correlated with the loss of body mass within the last 2 days of the experiment’s endpoint. Additionally, the phenylalanine level was also negatively correlated with muscle protein synthesis. QuanJun and collaborators [20] found elevated levels of creatine and 3-methylhistidine in the gastrocnemius muscle of tumour-bearing rats, suggesting a direct correlation between muscle wasting and intense protein catabolism. In another study, Cui and collaborators found increased tyrosine, phenylalanine, and methylhistidine levels in the gastrocnemius of a murine model of gastric cancer, suggesting that these metabolites were involved in intense metabolic wasting [39]. Some studies showed that π-methylhistidine and τ-methylhistidine were directly related to muscle protein degradation [40,41,42]. In our study, despite having no significant alteration in τ-methylhistidine, the muscle π-methylhistidine concentration in WW was also elevated (up to 3.3 times, as a trend) in all tumour evolution stages, showing that tumour growth can directly affect the skeletal muscle [43,44], pointing, in the initial tumour stage, to the start of proteolysis in weanling animals. However, in the young adult group, π-methylhistidine increased only in the AWi (threefold change, as a trend), likely showing the onset of muscle proteolysis. Despite the decreasing muscle π-methylhistidine content in the AWm and AWa groups, some previous studies showed an increased serum content of this amino acid derivative during the intense catabolic process, as previously reported [45,46,47]. Therefore, the specific difference in metabolism in weanlings compared with young adult animals could be the result of the discrepancy in the content of this amino acid [48,49]. On the other hand, this difference may be related to the muscle amino acid transport system, which could have been affected by tumour effects [50,51]. Thus, this comparison between ages likely showed severe spoliation in the WW, starting earlier than in the AW animals.

Glutamate and glutamine decreased in WW animals with tumour growth but remained the same in the AW animals. These amino acids are related to TCA as an intermediate precursor to ATP production or as a carbon precursor to the synthesis of nucleotides, and the interorgan metabolism of nitrogen and carbon metabolism [52]. In addition, glycine levels, which differed with the age of the animals, could be related to the synthesis of creatine (as seen here, an increased content in parallel with tumour evolution), porphyrins, and glutathione They are also involved in the aminoacyl-tRNA biosynthesis and nitrogen metabolism pathways, likely confirming protein spoliation in the muscle mass, especially in weanling animals, and especially because of the changed amino acid profiles in both tumour-bearing animals, due to the tumour’s growth needs and also the harmful effects of tumour evolution [53,54]. These data corroborate the trend of π-methylhistidine increase, which corresponds directly to myosin and actin degradation in skeletal muscle, already indicated as a cachexia biomarker [15,45,46,47]. In parallel, these two amino acids were related to the upregulation of glyoxylate and dicarboxylate metabolism, where the reduction in glycine was in association with the increase in creatine, suggesting an intense catabolism process, especially in weanling hosts.

Another point to discuss regarding the metabolomics results is the reduction of carnosine in WWi rats, which may be associated with possible targeting of this metabolite by the tumour. Recent research pointed out the potential physiological effects of carnosine in all host tissues and also in cancer cells [55]. Carnosine is related to the ability to inhibit glycolysis in tumour cells by its carbonyl quenching ability, reducing the generation of ATP in these cells [55]. In an exploratory view, a study by Yang and colleagues [15] observed a slight reduction in carnosine in the tumour-bearing group. Reduced carnosine could be related to an anti-neoplastic action; although, in our work, we verified intense tumour growth independent of the host’s age, even observing a significant increase in carnosine in AW compared with WW rats during tumour evolution.

Considering the energetic metabolism in cachectic muscle tissue, the provision of ATP for muscle contraction and activity can be completely altered, diverting this energy to the protein catabolic process, since anabolic capacity in this state is jeopardised [4]. Therefore, the energy requirements of skeletal muscle can be increased, which demands an enhanced flux of glycolysis, TCA cycling, and oxidative phosphorylation, which are jeopardised due to tumour effects [4,45,56,57]. In this way, the TCA cycle uses other intermediate metabolites such as amino acids and fatty acids [58], which resulted here in some metabolites interfering with the whole process. Before pyruvic acid can enter the TCA cycle, it is converted to acetyl-CoA to supply energy but can also provide many intermediate required for the synthesis of amino acids, glucose, haem, and others.

Some tissues, such as skeletal muscle, have a limited fatty acid synthase activity, which generates cytoplasm malonyl-CoA via acetyl-CoA carboxylase to regulate fatty acid oxidation [59,60]. On the other hand, in the muscle glycolytic process, the oxidation of glucose and fatty acids forms acetyl-CoA, which is then oxidised in the TCA, producing ATP [58]. In skeletal muscle, Abdel-aleem and colleagues stated that acetyl-CoA generated from glucose oxidation regulates fatty acid oxidation to guarantee energy production [61]. In addition, as seen especially in WW rats, the amino acids glycine, glutamate, alanine, and arginine, after deamination, could give rise to intermediate metabolites in the TCA [62]. However, the decreasing content of muscle succinate in WW rats and the slight reduction in AW groups could be related to muscle mitochondrial failure in energy production, as succinate is not available to be oxidised to fumarate (which was maintained in both the weanling and young adult groups), likely implicated in the lower action of succinate dehydrogenase, which is also part of the electron transport chain, as Complex II in the mitochondrial process [63,64,65]. Indeed, in our previous report, we found that the muscles’ mitochondrial function and the production of muscle ATP were reduced by the tumour’s effects [45]. In parallel, we reinforced the less efficient production of energy by some metabolites that point to this process, such as lactate. In the cachexia state, due to deficient energy production, fasting or stressed muscle releases the glycogen stores or, under low ATP content, diverts the glucose uptake to lactate, increasing the levels [64]. Our results showed maintenance of muscle lactate levels during tumour evolution. In some studies, there have been some controversial findings [36,39,64]. However, we suggest here that the intense glucose taken up by tumour cells or other tissues could decrease the muscle glucose supply, which led to maintaining the muscles’ lactate content.

**Figure 5:**
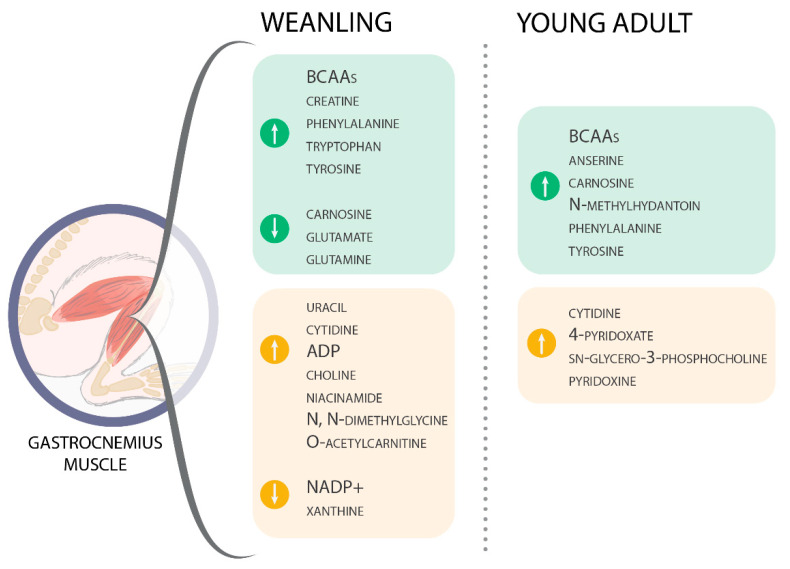
Main metabolic alterations identified in the gastrocnemius muscle in weanling and young adult Walker-256 tumour-bearing rats during cachexia evolution, leading to impacted metabolic pathways more related to an expressive catabolic process and diverted energy production in muscle tissue. In addition, the results showed the distinct impact of cancer cachexia regarding the host’s age.

In summary, the most significant variations found in our work, such as glutamate, glutamine, glycine, and methylhistidine, associated with succinate and cytidine, led us to point out some specific changes in the initial stage of tumour growth, which may be related to the start of muscle proteolysis and reduced production of energy by the muscle. All these metabolite changes were more expressive in the weanling hosts but they were also found in AW animals (e.g., carnosine, creatine, methylhydrantoin, and choline), and could also determine the onset of the cachexia state (Figure 5).

Some limitations of the present study may be raised for future investigations, especially regarding the translational value. First, the metabolomic extraction process and the technique/platform used here may differ from in some other studies, which could explain some of the different results found in the literature. In addition, the Walker-256 tumour, the experimental model used here, has an exponential growth that leads to a high tumour mass/body weight ratio, unlike in humans. Moreover, this study gave some insights into what happens at different stages of life but cannot predict what happens in paediatric cancer patients—the real translational ages used in this study [66]—as they do not suffer from cachexia or from this kind of tumour. Despite having these limitations, this study highlights the importance of using animals of the right age when studying cancer cachexia, since we showed here that the metabolism changes regarding age. The scientific community very often chooses the wrong animal age for performing preclinical studies relevant to many human pathologies [24], so the difference between these two animal ages shown here should be considered as proof of the need to improve future studies by adopting the right animal age in cancer cachexia studies.

## 4. Materials and Methods

### 4.1. Animals

Female Wistar rats at different ages were obtained from the Animal Facilities of the State University of Campinas, UNICAMP, Brazil. The general guidelines of the United Kingdom Coordinating Committee on Cancer Research (UKCCC, 1998) regarding animal welfare were followed. The 21-day-old animals were named “weanlings” (W) and the 90-day-old animals were named “young adults” (A), according to the developmental stages proposed by Sengupta [66]. The experimental protocol was approved by the Ethics Committee on Animal Experimentation of the Institute of Biology at the University of Campinas (CEUA; protocol number: #4918-1/2018; 5178-1/2019). All the animals were housed in individual cages under controlled environmental conditions (light and dark: 12/12 h; temperature: 22 ± 2 °C; humidity: 50–60%), were monitored daily, and given free access to food and water.

### 4.2. Experimental Protocol

The tumour-bearing animals were implanted with 2 × 10^6^ viable cells of the Walker-256 tumour (W) and were euthanised by decapitation at different states of tumour evolution: initial (WWi: 7 days, *n* = 9; AWi: 9 days, *n* = 5), intermediate (WWm: 9 days, *n* = 9; AWm: 14 days, *n* = 5), and advanced (WWa: 12–14 days, *n* = 13; AWa: 21 days, *n* = 5). The control groups were euthanised after 14 days for the weanling group (WC, *n* = 4) and after 21 days for the young adults (AC, *n* = 7). After the euthanasia, gastrocnemius muscles and tumours were resected and weighed. Fragments of gastrocnemius muscle were immediately frozen in liquid nitrogen and stored at −80 °C for further metabolomic analysis.

### 4.3. Nitrogen Balance

From each animal kept in individual metabolic cages, the nitrogen balance refers to nitrogen incorporation by food intake (mg N_2_ daily) minus the nitrogen excretion through faecal material (mg N_2_ in the total faecal mass in 24 h) and urinary excretion (mg N_2_ in the total volume of urine in 24 h). The diary nitrogen content in the diet, faeces, and urine were measured by using a colorimetric micro-Kjeldahl method. The results are presented as a percentage of mg N_2_ in 24 h vs. the initial value [67].

### 4.4. Metabolomic Analyses

#### 4.4.1. Muscle Sample Preparation for ^1^HNMR Acquisition

Gastrocnemius muscle samples were processed following Le Belle and colleagues’ protocol [68]. Briefly, frozen gastrocnemius muscle samples (≈100 mg) were added to a cold methanol/chloroform solution (2:1 *v*/*v*, in a total of 2.5 mL) and sonicated (VCX 500, Vibra-Cell; Sonics & Material Inc., Newtown, CT, USA) for 3 min with a 10 s pause between each minute. A cold chloroform/distilled water solution (1:1 *v*/*v*, total of 2.5 mL) was then added to the samples. Samples were briefly vortexed and centrifuged at 3000× *g* for 20 min at 4 °C. The polar phase was collected and dried in a vacuum concentrator (Vacufuge Concentrator; Eppendorf, Hamburg, Germany). The remaining solid phase was rehydrated in 0.6 mL of a D_2_O-containing phosphate buffer (0.1 M, pH 7.4) and 0.5 mM of TMSP-d4. The solution obtained was added to a 5 mm NMR tube for immediate acquisition.

#### 4.4.2. ^1^H-NMR Spectra Acquisition and Metabolic Quantification

The ^1^H-NMR spectra acquisition was performed using a Varian Inova NMR spectrometer (Agilent Technologies Inc., Santa Clara, CA, USA) equipped with a triple resonance probe and operating at a ^1^H resonance frequency of 500 MHz and a constant temperature of 298 K. In total, 256 free induction decays were collected with 32 K data points over a spectral width of 16 ppm. A 1.5 s relaxation delay was incorporated between scans, during which a continuous water pre-saturation radio frequency field was applied.

After data acquisition, spectroscopic data pre-processing was performed. Manual spectral processing, which included Fourier transformation, phasing correction, baseline correction, water region deletion, shim correction, apodisation (line broadening with lb ~0.3), and referencing control, was applied before the profiling was performed. The identification and quantification of the metabolites were made by computer-assisted manual fitting using Chenomx RMN Suite software (Chenomx Inc., Edmonton, Canada). To avoid bias, samples were randomly profiled blindly to the evaluator, and identified metabolites were fitted to each spectrum by the same human operator, resulting in sample profiles consisting of each metabolite. The results were normalised by tissue weight, and the metabolite concentrations are presented in millimoles per mg tissue.

### 4.5. Statistical Analysis

The morphometric parameters are presented as absolute and relative values. The delta body weight was calculated as (final body weight—initial body weight) for non-tumour-bearing groups and as ((final body weight-tumour weight)—initial body weight) for tumour-bearing groups. The relative values were calculated by dividing the gastrocnemius muscle and tumour weights by each animal’s respective initial body weight. The data analyses were performed by two-way ANOVA, followed by post-hoc Tukey’s honestly significant difference (HSD). Data are expressed as means ± standard deviation (SD) and *p* < 0.05 was considered to be significant. The gastrocnemius muscles’ metabolic profile data analyses were performed by two-way ANOVA, followed by post-hoc Tukey’s HSD. Data are expressed as means ± SD, and *p* < 0.05 was considered to be significant. The statistical analyses were performed using Graph Pad Prism 6.0 software (Graph-Pad Software, Inc.).

The metabolic pathways were analysed with a list of metabolites, which were significantly different in the comparisons by two-way ANOVA, followed by post-hoc Tukey’s HSD, by over-representation analysis, using the hypergeometric tests. Data are expressed as match status and regulation (upregulation or downregulation), and *p* < 0.05 adjusted by the false discovery rate (FDR) was considered significant. The statistical analyses were performed using the online MetaboAnalyst 4.0 platform (a statistical, functional, and integrative analysis of metabolomic data), more specifically the “Pathway Analysis” tool.

## Figures and Tables

**Table 1:** Metabolic pathways identified in weanling and young adult female Walker-256 tumour-bearing rats.

Pathway	Metabolite	Weanling			Young Adult			Weanling vs. Young Adult		
		Tumour Evolution			Tumour Evolution			Age		
		Match Status	Regulation	Adjusted *p*-Value *	Match Status	Regulation	Adjusted *p*-Value *	Match Status	Regulation	Adjusted *p*-Value *
Aminoacyl-tRNA biosynthesis	Glutamate	8/48	↓	**<0.01**	5/48	ns	**<0.01**	5/48	↑ vs. ↓	**0.01**
	Glutamine		↓			ns			↑ vs. ↓	
	Glycine		ns			ns			↑ vs. ↓	
	Histidine		ns			ns			↑ vs. ↓	
	Isoleucine		↑			↑			ns	
	Leucine		↑			↑			ns	
	Phenylalanine		↑			↑			ns	
	Tryptophan		↑			ns			↑ vs. ↓	
	Tyrosine		↑			↑			ns	
	Valine		↑			↑			ns	
Valine, leucine, and isoleucine biosynthesis	Isoleucine	3/8	↑	**<0.01**	3/8	↑	**<0.01**	0/8	ns	ne
	Leucine		↑			↑			ns	
	Valine		↑			↑			ns	
Phenylalanine, tyrosine, and tryptophan biosynthesis	Phenylalanine	2/4	↑	**0.02**	2/4	↑	**0.01**	0/4	ns	ne
	Tyrosine		↑			↑			ns	
Nitrogen metabolism	Glutamate	2/6	↓	**0.03**	0/6	ns	ne	2/6	↑ vs. ↓	**0.03**
	Glutamine		↓			ns			↑ vs. ↓	
D-glutamine and D-glutamate metabolism	Glutamate	2/6	↓	**0.03**	0/6	ns	ne	2/6	↑ vs. ↓	**0.03**
	Glutamine		↓			ns			↑ vs. ↓	
Vitamin B6 metabolism	4-Pyridoxate	0/9	ns	ne	2/9	↑	**0.04**	2/9	↓ vs. ↑	0.06
	Pyridoxine		ns			↑			↓ vs. ↑	
Histidine metabolism	Anserine	2/16	ns	0.11	2/16	↑	0.07	6/16	↓ vs. ↑	**<0.01**
	Carnosine		↓			↑			↓ vs. ↑	
	Glutamate		↓			ns			↑ vs. ↓	
	Histamine		ns			ns			↑ vs. ↓	
	Histidine		ns			ns			↑ vs. ↓	
	π-Methylhistidine		ns			ns			↑ vs. ↓	
β-Alanine metabolism	Anserine	2/21	ns	0.166	2/21	↑	0.11	4/21	↓ vs. ↑	**0.01**
	Carnosine		↓			↑			↓ vs. ↑	
	Histidine		ns			ns			↑ vs. ↓	
	Uracil		↑			ns			↑ vs. ↓	
Glyoxylate and dicarboxylate metabolism	Acetate	2/32	ns	0.27	0/32	ns	ne	4/32	↑ vs. ↓	**0.01**
	Glutamate		↓			ns			↑ vs. ↓	
	Glutamine		↓			ns			↑ vs. ↓	
	Glycine		ns			ns			↑ vs. ↓	

Weanling and young adult female rats were distributed by tumour evolution (control—WC and AC; initial—WWi and AWi; intermediate—WWm and AWm; advanced—WWa and AWa) and age (WC and AC, WWi and AWi, WWm and AWm, and WWa and AWa). Data are represented as match status and regulation, which were analysed by over-representation analysis through hypergeometric tests (comparing tumour evolution in weanling and young adults, and age in weanlings vs. young adults). *: *p*-value adjusted by the false discovery rate method. ↑ and ↓: up- and downregulation, respectively. ns and ne: no significance and not evaluated, respectively. Bold *p*-values represent a significant difference.

## Data Availability

The data presented in this study are available in article and Supplementary Material.

## Supplementary Materials

The following are available online at https://www.mdpi.com/article/10.3390/metabo11060404/s1, Figure S1: Overlap of one representative 500 MHz ^1^H-NMR spectrum of the gastrocnemius muscle aqueous fraction per group, Table S1: Morphometric parameters and N_2_ balance identified in weanling and young adult female Walker-256 tumour-bearing rats, Table S2: Total gastrocnemius muscle metabolic profile identified in weanling and young adult female Walker-256 tumour-bearing rats.
