# Supplementary material for: A Time-Course Comparison of Skeletal Muscle Metabolomic Alterations in Walker-256 Tumour-Bearing Rats at Different Stages of Life
Source: Metabolites. 2021 Jun 20;11(6):404. doi: 10.3390/metabo11060404 (PMC8234487; doi:10.3390/metabo11060404)
Supplement: Supplementary file 1 [file metabolites-11-00404-s001.zip › Chiocchetti et al Supplementary Tables R2.pdf]

Supplementary Table S1. Morphometric parameters and N_2 balance identified in weanling and young adult female Walker-256 tumour-bearing rats.

**Morphometric parameters and N_2 balance**	**Weanling**				**Young adult**				**F (DFn, DFd)**			***P*-value**		
	**Control**	**Initial**	**Intermediated**	**Advanced**	**Control**	**Initial**	**Intermediated**	**Advanced**	**Interaction**	**Tumour evolution**	**Age**	**Interaction**	**Tumour evolution**	**Age**
	**Mean ± SD**	**Mean ± SD**	**Mean ± SD**	**Mean ± SD**	**Mean ± SD**	**Mean ± SD**	**Mean ± SD**	**Mean ± SD**						
Initial weight (g)	51.25 ± 1.25	56.63 ± 5.67	60.47 ± 6.19	59.24 ± 3.98	256.16 ± 16.10	252.08 ± 11.91	241.45 ± 8.28	279.54 ± 9.95	11.89 (3, 45)	12.80 (3, 45)	6574.00 (1, 45)	**<0.01**	**<0.01**	**<0.01**
Final weight (g)	126.25 ± 2.63	85.35 ± 9.57	95.64 ± 10.36	115.11 ± 20.49	268.65 ± 14.88	254.50 ± 8.97	256.87 ± 6.95	282.37 ± 26.30	1.60 (3, 49)	11.31 (3, 49)	1239.00 (1, 49)	0.20	**<0.01**	**<0.01**
Δ Body weight (g)	75.00 ± 1.41	27.44 ± 5.27	32.19 ± 7.65	43.48 ± 14.22	12.48 ± 5.25	-9.90 ± 11.14	-12.90 ± 8.78	-19.38 ± 30.86	2.98 (3, 45)	17.22 (3, 45)	185.90 (1, 45)	**0.04**	**<0.01**	**<0.01**
Tumour weight (g)	--	1.27 ± 0.83	3.26 ± 1.92	8.52 ± 3.07	--	12.32 ± 2.39	28.32 ± 6.01	32.28 ± 13.81	5.65 (2.41)	20.19 (2, 41)	115.60 (1, 41)	**0.01**	**<0.01**	**<0.01**
Tumour relative weight (g)	--	0.02 ± 0.01	0.06 ± 0.03	0.14 ± 0.05	--	0.05 ± 0.01	0.12 ± 0.03	0.12 ± 0.05	3.78 (2, 39)	21.05 (2, 39)	2.96 (1, 39)	**0.03**	**<0.01**	0.09
Gastrocnemius weight (g)	0.74 ± 0.02	0.45 ± 0.04	0.47 ± 0.05	0.44 ± 0.12	1.93 ± 0.14	1.56 ± 0.45	1.22 ± 0.17	1.22 ± 0.36	3.50 (3, 49)	14.70 (3, 49)	268.30 (1, 49)	**0.02**	**<0.01**	**<0.01**
Gastrocnemius relative weight (g)	1.45 ± 0.02	0.81 ± 0.11	0.81 ± 0.15	0.70 ± 0.19	0.75 ± 0.05	0.62 ± 0.18	0.50 ± 0.05	0.45 ± 0.15	6.60 (3, 45)	27.25 (3, 45)	71.52 (1, 45)	**<0.01**	**<0.01**	**<0.01**
Food intake N_2 (mg)	188.27 ± 33.52	146.97 ± 54.72	114.29 ± 62.83	37.98 ± 30.79	715.59 ± 202.79	401.73 ± 97.31	332.63 ± 85.55	139.00 ± 92.07	20.17 (3, 76)	56.06 (3, 76)	188.10 (1,76)	**<0.01**	**<0.01**	**<0.01**
Urinary N_2 (mg)	38.54 ± 28.61	47.13 ± 19.07	31.57 ± 17.50	16.22 ± 19.75	159.30 ± 69.63	181.23 ± 59.18	86.00 ± 30.78	127.44 ± 74.37	3.45 (3, 76)	7.11 (3, 76)	124.00 (1, 76)	**0.02**	**<0.01**	**<0.01**
Faecal N_2 (mg)	19.19 ± 8.02	8.61 ± 5.72	6.00 ± 2.93	5.02 ± 3.55	33.54 ± 21.84	35.93 ± 14.14	25.35 ± 8.84	18.76 ± 12.65	1.78 (3, 76)	7.65 (3, 76)	63.36 (1, 76)	0.15	**<0.01**	**<0.01**

N_2 balance (mg)	130.54 ± 35.48	101.17 ± 29.05	90.27 ± 37.94	16.75 ± 29.76	522.75 ± 169.80	184.57 ± 78.24	221.28 ± 76.11	-7.21 ± 45.68	30.48 (3, 74)	68.01 (3, 74)	81.43 (1, 74)	**<0.01**	**<0.01**	**<0.01**
N_2 balance (%)	100.00 ± 27.18	77.50 ± 22.25	69.15 ± 29.07	12.83 ± 22.79	100.00 ± 32.48	35.31 ± 14.97	42.33 ± 14.56	-1.38 ± 8.74	3.03 (3, 74)	56.82 (3,74)	16.25 (1, 74)	**0.03**	**<0.01**	**<0.01**

Legend: Weanling and young adult female rats were distributed into control (WC and AC) and initial (WWi and AWi), intermediated (WWm and AWm) and advanced (WWa and AWa) Walker tumour-bearing. DFn and DFd: degrees of freedom for the numerator and denominator of the F ratio, respectively. Data were expressed as mean ± standard deviation (SD) and analysed by two-way ANOVA. Bold *P* values represented a significant difference.

Supplementary Table S2. Total gastrocnemius muscle metabolic profile identified in weanling and young adult female Walker-256 tumour-bearing rats.

**Metabolite**	**Weanling**				**Young adult**				**F (DFn, DFd)**			***P*-value**		
	**Control**	**Initial**	**Intermediated**	**Advanced**	**Control**	**Initial**	**Intermediated**	**Advanced**						
	**Mean ± SD**	**Mean ± SD**	**Mean ± SD**	**Mean ± SD**	**Mean ± SD**	**Mean ± SD**	**Mean ± SD**	**Mean ± SD**	**Interaction**	**Tumour evolution**	**Age**	**Interaction**	**Tumour evolution**	**Age**
4-Pyridoxate	4.60 ± 1.40	3.73 ± 1.72	5.32 ± 2.82	7.60 ± 4.18	5.00 ± 2.52	9.27 ± 1.66	6.33 ± 2.94	18.92 ± 8.87	5.83 (3, 48)	13.53 (3, 48)	19.15 (1, 48)	**<0.01**	**<0.01**	**<0.01**
Acetate	7.75 ± 2.33	10.42 ± 4.31	12.17 ± 4.75	13.66 ± 4.25	6.67 ± 1.60	10.32 ± 3.39	5.35 ± 2.32	6.40 ± 1.10	3.38 (3, 49)	1.77 (3, 49)	13.57 (1, 49)	**0.03**	0.17	**<0.01**
ADP	9.20 ± 7.14	10.97 ± 3.86	22.25 ± 7.90	14.82 ± 7.20	8.87 ± 5.07	8.03 ± 1.13	7.51 ± 2.67	8.32 ± 4.24	3.37 (3, 48)	2.48 (3, 48)	13.08 (1, 48)	**0.03**	0.07	**<0.01**
Alanine	282.40 ± 90.89	278.28 ± 61.40	256.73 ± 90.73	281.50 ± 127.26	129.36 ± 24.02	148.33 ± 17.55	139.94 ± 31.63	193.21 ± 41.29	0.33 (3, 49)	0.58 (3, 49)	27.32 (1, 49)	0.80	0.63	**<0.01**
AMP	349.56 ± 105.97	302.10 ± 74.54	317.03 ± 127.16	315.61 ± 130.52	254.13 ± 76.33	328.20 ± 19.52	323.41 ± 96.75	430.20 ± 88.13	2.11 (3, 49)	1.22 (3, 49)	0.20 (1, 49)	0.11	0.31	0.66
Anserine	62.91 ± 44.75	84.07 ± 28.58	102.18 ± 14.38	89.37 ± 43.63	99.66 ± 62.67	188.39 ± 74.52	258.12 ± 191.55	256.59 ± 99.99	1.54 (3, 46)	3.62 (3, 46)	26.54 (1, 46)	0.21	**0.02**	**<0.01**
Carnosine	490.07 ± 160.53	264.40 ± 78.46	429.98 ± 92.40	317.31 ± 98.55	423.20 ± 113.28	474.55 ± 42.27	271.23 ± 158.08	700.95 ± 166.17	15.80 (3, 48)	5.68 (3, 48)	8.38 (1, 48)	**<0.01**	**<0.01**	**<0.01**
Choline	11.17 ± 2.77	13.35 ± 3.37	21.39 ± 4.64	27.45 ± 11.29	33.70 ± 11.79	26.12 ± 7.86	25.47 ± 16.73	26.52 ± 5.45	3.17 (3, 47)	1.14 (3, 47)	11.96 (1, 47)	**0.03**	0.34	**<0.01**
Creatine	2035.82 ± 660.75	1644.58 ± 487.87	2264.31 ± 570.31	2505.65 ± 684.57	1508.52 ± 367.35	1934.75 ± 242.49	1830.90 ± 565.41	2390.04 ± 480.99	1.38 (3, 49)	4.38 (3, 49)	1.60 (1, 49)	0.26	**0.01**	0.21
Cytidine	5.23 ± 1.57	2.97 ± 0.87	4.55 ± 1.17	6.86 ± 3.01	1.17 ± 0.22	1.87 ± 0.25	4.02 ± 2.13	5.61 ± 2.41	1.90 (3, 49)	10.31 (3, 49)	10.39 (1, 49)	0.14	**<0.01**	**<0.01**

Formate	3.33 ± 1.09	3.24 ± 3.34	3.93 ± 2.08	5.93 ± 2.23	2.36 ± 0.81	3.19 ± 2.05	2.68 ± 1.48	2.51 ± 0.87	1.57 (3, 49)	0.99 (3, 49)	5.61 (1, 49)	0.21	0.41	**0.02**
Fumarate	6.32 ± 1.96	3.55 ± 1.01	4.85 ± 1.99	5.66 ± 2.12	3.15 ± 0.89	2.95 ± 1.11	2.46 ± 0.89	3.10 ± 0.63	1.53 (3, 49)	2.22 (3, 49)	24.05 (1, 49)	0.22	0.10	**<0.01**
Glucuronate	80.69 ± 17.25	109.18 ± 20.21	131.19 ± 91.04	133.26 ± 141.76	97.96 ± 64.96	129.48 ± 13.64	144.37 ± 39.95	182.38 ± 35.53	0.12 (3, 48)	1.25 (3, 48)	0.99 (1, 48)	0.94	0.30	0.32
Glutamate	160.08 ± 49.97	168.54 ± 55.07	91.91 ± 40.78	84.62 ± 31.19	41.81 ± 14.57	62.41 ± 15.03	52.51 ± 28.71	78.96 ± 22.49	6.97 (3, 49)	3.67 (3, 49)	42.87 (1, 49)	**<0.01**	**0.02**	**<0.01**
Glutamine	347.71 ± 71.82	272.07 ± 86.09	182.32 ± 107.58	131.51 ± 75.39	98.90 ± 37.26	126.15 ± 19.58	82.26 ± 17.53	97.63 ± 44.92	4.16 (3, 48)	5.96 (3, 48)	39.08 (1, 48)	**0.01**	**<0.01**	**<0.01**
Glycine	306.49 ± 116.97	372.54 ± 175.83	318.74 ± 179.18	217.11 ± 152.58	90.47 ± 19.07	118.03 ± 10.07	80.60 ± 19.53	139.28 ± 27.95	1.33 (3, 49)	0.63 (3, 49)	28.20 (1, 49)	0.27	0.60	**<0.01**
Histamine	2.17 ± 0.67	1.63 ± 0.80	1.58 ± 0.81	2.54 ± 1.04	0.65 ± 0.41	1.09 ± 0.48	0.70 ± 0.21	0.79 ± 0.20	1.83 (3, 49)	1.14 (3, 49)	30.81 (1, 49)	0.15	0.34	**<0.01**
Histidine	1.88 ± 0.59	1.28 ± 0.60	1.02 ± 0.48	1.83 ± 1.74	0.37 ± 0.36	0.33 ± 0.20	0.28 ± 0.14	0.18 ± 0.05	0.71 (3, 49)	0.61 (3, 49)	20.65 (1, 49)	0.55	0.61	**<0.01**
Imidazole	1.06 ± 0.40	1.09 ± 0.39	1.42 ± 0.56	1.54 ± 0.43	1.53 ± 0.80	1.32 ± 0.81	0.85 ± 0.41	1.10 ± 0.38	2.64 (3, 49)	0.32 (3, 49)	0.27 (1, 49)	0.06	0.81	0.60
Inosine	12.75 ± 4.18	9.73 ± 2.88	12.50 ± 4.48	15.92 ± 7.77	13.50 ± 4.95	19.12 ± 6.27	14.45 ± 3.67	20.91 ± 4.13	1.54 (3, 49)	2.73 (3, 49)	7.74 (1, 49)	0.22	0.05	**0.01**
Isoleucine	6.11 ± 1.96	6.52 ± 2.13	8.58 ± 3.24	11.24 ± 3.19	5.12 ± 1.24	7.38 ± 1.27	7.86 ± 3.39	11.81 ± 3.88	0.34 (3, 49)	10.74 (3, 49)	0.01 (1, 49)	0.80	**<0.01**	0.93
Lactate	1997.95 ± 687.93	1950.12 ± 737.34	2664.32 ± 1108.03	2692.80 ± 981.74	2356.89 ± 701.73	3368.42 ± 415.28	2619.18 ± 820.11	3705.32 ± 582.62	1.92 (3, 49)	3.02 (3, 49)	8.20 (1, 49)	0.14	**0.04**	**0.01**

Leucine	10.66 ± 3.96	11.85 ± 3.68	15.05 ± 3.80	23.90 ± 7.08	7.44 ± 1.77	10.51 ± 1.99	11.69 ± 3.76	16.23 ± 4.60	1.16 (3, 49)	13.82 (3, 49)	8.78 (1, 49)	0.34	**<0.01**	**<0.01**
Lysine	144.78 ± 55.63	131.18 ± 67.08	151.64 ± 82.74	139.42 ± 75.30	52.15 ± 17.70	59.46 ± 4.81	61.30 ± 24.56	90.69 ± 30.74	0.38 (3, 49)	0.29 (3, 49)	19.81 (1, 49)	0.77	0.83	**<0.01**
Maltose	8.30 ± 5.18	8.55 ± 3.37	7.47 ± 5.16	12.47 ± 15.69	5.13 ± 1.86	8.37 ± 5.19	7.78 ± 3.10	6.02 ± 0.60	0.35 (3, 47)	0.16 (3, 47)	0.84 (1, 47)	0.78	0.92	0.36
Methionine	7.07 ± 2.15	8.28 ± 3.43	7.40 ± 2.82	9.26 ± 1.91	4.50 ± 2.11	7.48 ± 3.59	6.82 ± 3.25	8.27 ± 3.39	0.29 (3, 49)	2.44 (3, 49)	2.44 (1, 49)	0.83	0.08	0.12
myo-Inositol	37.35 ± 14.26	45.32 ± 7.86	50.78 ± 14.68	43.84 ± 15.64	15.68 ± 4.03	15.57 ± 3.57	21.95 ± 9.67	20.84 ± 6.90	0.34 (3, 47)	1.41 (3, 47)	55.06 (1, 47)	0.79	0.24	**<0.01**
N,N-Dimethylglycine	5.06 ± 1.62	4.07 ± 1.12	5.83 ± 1.75	6.06 ± 1.46	1.60 ± 1.57	1.47 ± 1.25	1.05 ± 1.13	0.61 ± 0.31	2.82 (3, 49)	0.61 (3, 49)	106.60 (1, 49)	0.05	0.61	**<0.01**
NAD+	13.98 ± 4.61	9.89 ± 3.63	9.85 ± 4.14	5.52 ± 4.15	6.56 ± 7.69	3.17 ± 1.16	3.19 ± 2.36	2.99 ± 0.72	0.88 (3, 48)	3.50 (3, 48)	21.67 (1, 48)	0.45	**0.02**	**<0.01**
NADP+	14.83 ± 4.71	11.38 ± 4.09	10.02 ± 4.07	4.48 ± 2.91	0.99 ± 0.48	1.03 ± 0.71	1.44 ± 1.50	0.62 ± 0.10	4.32 (3, 46)	5.06 (3, 46)	96.23 (1, 46)	**<0.01**	**<0.01**	**<0.01**
Niacinamide	13.18 ± 4.09	13.51 ± 6.35	16.64 ± 7.28	24.87 ± 7.78	14.60 ± 4.47	20.33 ± 5.58	20.69 ± 8.67	26.77 ± 5.50	0.42 (3, 49)	7.39 (3, 49)	3.49 (1, 49)	0.74	**<0.01**	0.07
Nicotinurate	3.35 ± 1.57	5.16 ± 2.92	6.73 ± 3.18	8.24 ± 5.65	8.31 ± 2.64	8.49 ± 2.65	10.36 ± 2.43	11.35 ± 2.52	0.14 (3, 49)	2.87 (3, 49)	13.03 (1, 49)	0.93	0.05	**<0.01**
N-Methylhydantoin	3.25 ± 1.08	2.96 ± 1.81	3.99 ± 1.19	4.78 ± 1.42	10.04 ± 2.95	11.14 ± 0.93	11.56 ± 3.69	14.43 ± 2.67	1.12 (3, 49)	5.44 (3, 49)	192.90 (1, 49)	0.35	**<0.01**	**<0.01**
O-Acetylcarnitine	9.23 ± 3.02	15.80 ± 5.21	10.39 ± 3.51	17.76 ± 5.24	12.38 ± 3.31	11.34 ± 3.89	12.13 ± 6.41	12.02 ± 2.32	3.01 (3, 49)	2.35 (3, 49)	1.08 (1, 49)	**0.04**	0.08	0.30

Phenylalanine	4.80 ± 1.44	4.99 ± 1.61	8.83 ± 2.50	12.63 ± 3.98	5.53 ± 1.51	9.09 ± 1.25	9.38 ± 3.63	13.94 ± 4.63	0.99 (3, 49)	17.83 (3, 49)	3.99 (1, 49)	0.41	**<0.01**	0.05
Pyridoxine	2.66 ± 0.80	0.89 ± 0.65	2.16 ± 1.48	1.51 ± 0.69	2.33 ± 1.77	3.40 ± 0.59	4.16 ± 3.22	6.06 ± 3.96	3.85 (3, 49)	2.26 (3, 49)	18.90 (1, 49)	**0.01**	0.09	**<0.01**
sn-Glycero-3-phosphocholine	8.85 ± 2.48	8.82 ± 4.56	7.95 ± 2.72	10.36 ± 2.36	9.29 ± 2.25	14.17 ± 3.23	17.61 ± 5.66	15.81 ± 4.89	3.01 (3, 46)	2.56 (3, 46)	24.16 (1, 46)	**0.04**	0.07	**<0.01**
Succinate	60.01 ± 24.16	40.94 ± 24.22	32.80 ± 14.69	29.57 ± 21.75	11.56 ± 11.53	2.96 ± 0.99	3.35 ± 1.79	4.30 ± 1.34	1.03 (3, 49)	2.78 (3, 49)	51.52 (1, 49)	0.39	0.05	**<0.01**
Taurine	1005.47 ± 174.86	667.40 ± 176.18	978.88 ± 252.78	894.52 ± 291.76	760.44 ± 225.21	1036.75 ± 202.74	901.59 ± 162.11	1068.94 ± 271.30	3.87 (3, 49)	0.79 (3, 49)	0.68 (1, 49)	**0.01**	0.50	0.41
Theophylline	1.51 ± 1.02	2.62 ± 2.43	1.56 ± 1.01	1.95 ± 1.23	0.88 ± 0.30	1.80 ± 1.16	0.87 ± 0.35	2.41 ± 1.27	0.66 (3, 49)	2.23 (3, 49)	1.19 (1, 49)	0.58	0.10	0.28
Tryptophan	2.03 ± 0.63	2.16 ± 0.59	3.05 ± 1.04	3.38 ± 1.19	1.27 ± 0.30	1.58 ± 0.39	1.40 ± 0.26	2.30 ± 1.02	0.97 (3, 49)	4.84 (3, 49)	17.99 (1, 49)	0.42	**0.01**	**<0.01**
Tyrosine	9.39 ± 2.82	8.60 ± 2.38	11.99 ± 3.45	15.98 ± 6.66	5.95 ± 1.31	9.81 ± 1.24	10.24 ± 5.88	14.46 ± 5.28	0.55 (3, 49)	6.98 (3, 49)	1.19 (1, 49)	0.65	**<0.01**	0.28
UDP-glucuronate	12.25 ± 5.34	7.46 ± 3.63	10.74 ± 3.59	13.12 ± 6.83	4.33 ± 1.15	7.49 ± 1.62	8.25 ± 5.90	13.02 ± 5.95	1.64 (3, 49)	3.52 (3, 49)	3.62 (1, 49)	0.19	**0.02**	0.06
UDP-N-Acetylglucosamine	5.13 ± 4.03	4.00 ± 1.93	5.85 ± 3.52	3.80 ± 2.47	1.45 ± 0.36	1.66 ± 0.71	3.80 ± 4.83	2.79 ± 3.47	0.44 (3, 49)	1.21 (3, 49)	7.91 (3, 49)	0.72	0.32	**0.01**
Uracil	2.58 ± 1.18	2.26 ± 0.54	2.67 ± 0.88	4.64 ± 2.46	0.57 ± 0.25	1.14 ± 0.29	1.07 ± 0.47	1.38 ± 0.22	1.63 (3, 49)	3.26 (3, 49)	27.88 (1, 49)	0.19	**0.03**	**<0.01**
Valine	12.15 ± 3.80	13.83 ± 2.97	15.49 ± 3.08	19.80 ± 5.08	8.82 ± 1.91	12.26 ± 3.03	11.68 ± 4.45	16.06 ± 3.68	0.25 (3, 49)	8.57 (3, 49)	8.51 (1, 49)	0.86	**<0.01**	**0.01**

Xanthine	6.22 ± 2.01	3.26 ± 1.89	1.79 ± 1.01	4.56 ± 2.44	1.12 ± 0.30	2.27 ± 1.27	0.91 ± 0.20	0.79 ± 0.32	4.97 (3, 49)	4.12 (3, 49)	34.42 (1, 49)	**<0.01**	**0.01**	**<0.01**
π-Methylhistidine	8.64 ± 5.82	24.43 ± 13.20	20.53 ± 6.79	21.61 ± 13.86	6.29 ± 4.16	19.10 ± 6.18	2.23 ± 1.98	1.25 ± 0.21	2.58 (3, 48)	4.73 (3, 48)	16.99 (1, 48)	0.06	**<0.01**	**<0.01**
τ-Methylhistidine	2.65 ± 3.14	1.93 ± 1.02	2.27 ± 0.66	1.61 ± 0.71	1.23 ± 0.59	1.10 ± 0.83	1.33 ± 0.82	1.15 ± 0.80	0.39 (3, 49)	0.67 (3, 49)	8.80 (1, 49)	0.76	0.57	**<0.01**

Legend: Weanling and young adult female rats were distributed into control (WC and AC) and initial (WWi and AWi), intermediated (WWm and AWm) and advanced (WWa and AWa) Walker tumour-bearing. DFn and DFd: degrees of freedom for the numerator and denominator of the F ratio, respectively. Data were expressed as mean ± standard deviation (SD) (mM/ mg tissue) and analysed by two-way ANOVA. Bold *P* values represented a significant difference.
