# Supplementary figures and images for: A Time-Course Comparison of Skeletal Muscle Metabolomic Alterations in Walker-256 Tumour-Bearing Rats at Different Stages of Life
Source: Metabolites. 2021 Jun 20;11(6):404. doi: 10.3390/metabo11060404 (PMC8234487; doi:10.3390/metabo11060404)

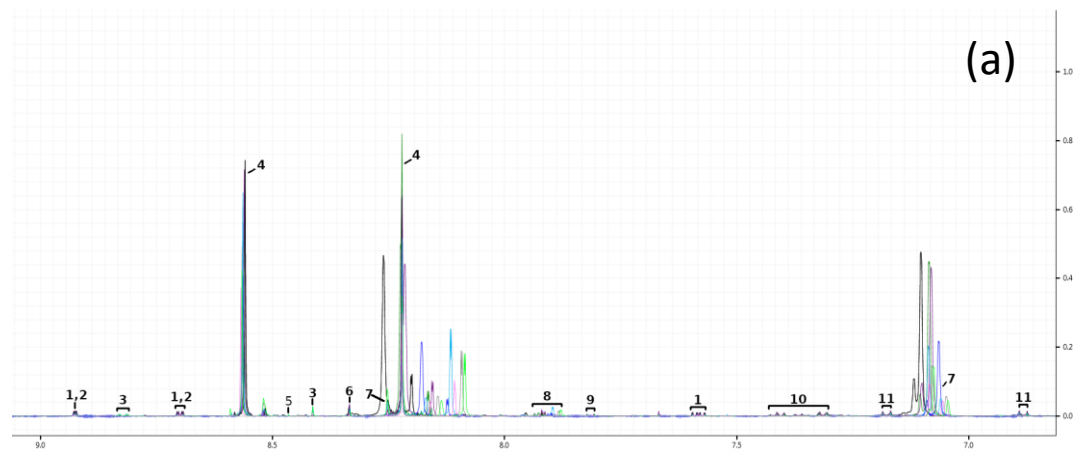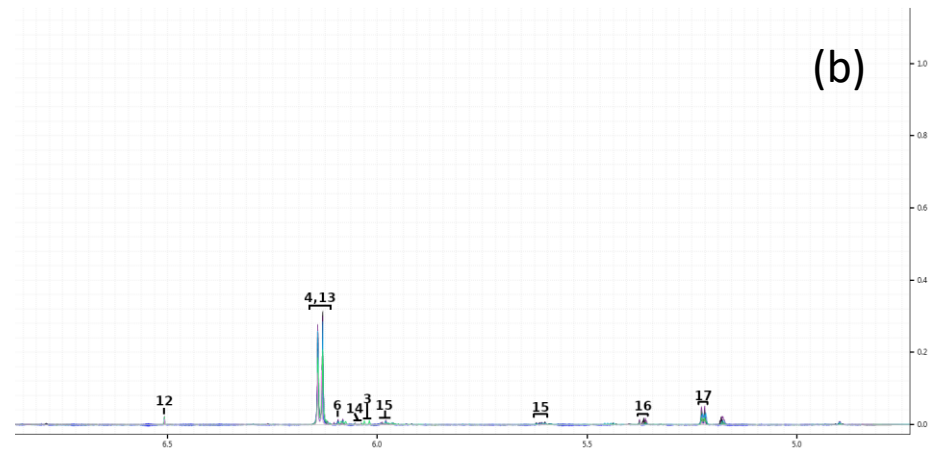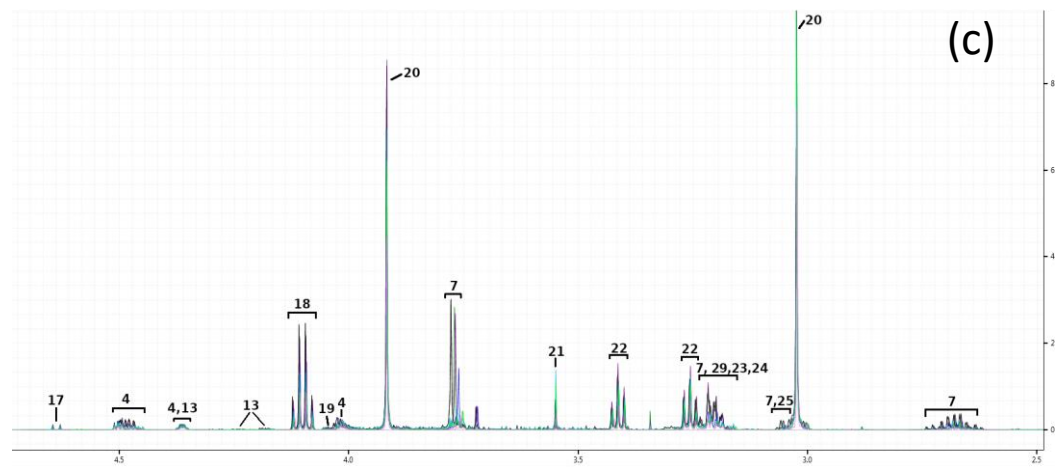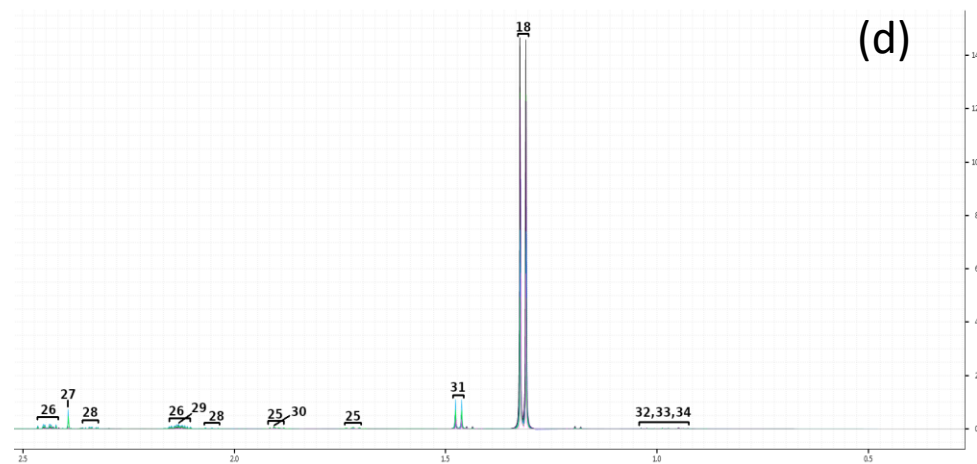

Supplement: Supplementary file 1 [file metabolites-11-00404-s001.zip › Chiocchetti et al Figure S1 (1).pdf]
